# Conflicts of Interest at Medical Journals: The Influence of Industry-Supported Randomised Trials on Journal Impact Factors and Revenue – Cohort Study

**DOI:** 10.1371/journal.pmed.1000354

**Published:** 2010-10-26

**Authors:** Andreas Lundh, Marija Barbateskovic, Asbjørn Hróbjartsson, Peter C. Gøtzsche

**Affiliations:** 1 The Nordic Cochrane Centre, Rigshospitalet, Copenhagen, Denmark; 2 Institute of Medicine and Surgery, Faculty of Health Sciences, University of Copenhagen, Copenhagen, Denmark

## Abstract

**Background:**

Transparency in reporting of conflict of interest is an increasingly important aspect of publication in medical journals. Publication of large industry-supported trials may generate many citations and journal income through reprint sales and thereby be a source of conflicts of interest for journals. We investigated industry-supported trials' influence on journal impact factors and revenue.

**Methods and Findings:**

We sampled six major medical journals (*Annals of Internal Medicine*, *Archives of Internal Medicine*, *BMJ*, *JAMA*, The *Lancet*, and *New England Journal of Medicine* [*NEJM*]). For each journal, we identified randomised trials published in 1996–1997 and 2005–2006 using PubMed, and categorized the type of financial support. Using Web of Science, we investigated citations of industry-supported trials and the influence on journal impact factors over a ten-year period. We contacted journal editors and retrieved tax information on income from industry sources. The proportion of trials with sole industry support varied between journals, from 7% in *BMJ* to 32% in *NEJM* in 2005–2006. Industry-supported trials were more frequently cited than trials with other types of support, and omitting them from the impact factor calculation decreased journal impact factors. The decrease varied considerably between journals, with 1% for *BMJ* to 15% for *NEJM* in 2007. For the two journals disclosing data, income from the sales of reprints contributed to 3% and 41% of the total income for *BMJ* and The *Lancet* in 2005–2006.

**Conclusions:**

Publication of industry-supported trials was associated with an increase in journal impact factors. Sales of reprints may provide a substantial income. We suggest that journals disclose financial information in the same way that they require them from their authors, so that readers can assess the potential effect of different types of papers on journals' revenue and impact.

*Please see later in the article for the Editors' Summary*

## Abstract 2

Andreas Lundh and colleagues investigated the effect of publication of large industry-supported trials on citations and journal income, through reprint sales, in six general medical journals

## Editors' Summary

**Background:**

Medical journals publish many different types of papers that inform doctors about the latest research advances and the latest treatments for their patients. They publish articles that describe laboratory-based research into the causes of diseases and the identification of potential new drugs. They publish the results of early clinical trials in which a few patients are given a potential new drug to check its safety. Finally and most importantly, they publish the results of randomized controlled trials (RCTs). RCTs are studies in which large numbers of patients are randomly allocated to different treatments without the patient or the clinician knowing the allocation and the efficacy of the various treatments compared. RCTs are best way of determining whether a new drug is effective and have to be completed before a drug can be marketed. Because RCTs are very expensive, they are often supported by drug companies. That is, drug companies provide grants or drugs for the trial or assist with data analysis and/or article preparation.

**Why Was This Study Done?:**

Whenever a medical journal publishes an article, the article's authors have to declare any conflicts of interest such as financial gain from the paper's publication. Conflict of interest statements help readers assess papers—an author who owns the patent for a drug, for example, might put an unduly positive spin on his/her results. The experts who review papers for journals before publication provide similar conflict of interest statements. But what about the journal editors who ultimately decide which papers get published? The International Committee of Medical Journal Editors (ICMJE), which produces medical publishing guidelines, states that: “Editors who make final decisions about manuscripts must have no personal, professional, or financial involvement in any of the issues that they might judge.” However, the publication of industry-supported RCTs might create “indirect” conflicts of interest for journals by boosting the journal's impact factor (a measure of a journal's importance based on how often its articles are cited) and its income through the sale of reprints to drug companies. In this study, the researchers investigate whether the publication of industry-supported RCTs influences the impact factors and finances of six major medical journals.

**What Did the Researchers Do and Find?:**

The researchers determined which RCTs published in the *New England Journal of Medicine* (*NEJM*), the *British Medical Journal* (*BMJ*), The *Lancet*, and three other major medical journals in 1996–1997 and 2005–2006 were supported wholly, partly, or not at all by industry. They then used the online academic citation index Web of Science to calculate an approximate impact factor for each journal for 1998 and 2007 and calculated the effect of the published RCTs on the impact factor. The proportion of RCTs with sole industry support varied between journals. Thus, 32% of the RCTs published in the *NEJM* during both two-year periods had industry support whereas only 7% of the RCTs published in the *BMJ* in 2005–2006 had industry support. Industry-supported trials were more frequently cited than RCTs with other types of support and omitting industry-supported RCTs from impact factor calculations decreased all the approximate journal impact factors. For example, omitting all RCTs with industry or mixed support decreased the 2007 *BMJ* and *NEJM* impact factors by 1% and 15%, respectively. Finally, the researchers asked each journal's editor about their journal's income from industry sources. For the *BMJ* and The *Lancet*, the only journals that provided this information, income from reprint sales was 3% and 41%, respectively, of total income in 2005–2006.

**What Do These Findings Mean?:**

These findings show that the publication of industry-supported RCTs was associated with an increase in the approximate impact factors of these six major medical journals. Because these journals publish numerous RCTs, this result may not be generalizable to other journals. These findings also indicate that income from reprint sales can be a substantial proportion of a journal's total income. Importantly, these findings do not imply that the decisions of editors are affected by the possibility that the publication of an industry-supported trial might improve their journal's impact factor or income. Nevertheless, the researchers suggest, journals should live up to the same principles related to conflicts of interest as those that they require from their authors and should routinely disclose information on the source and amount of income that they receive.

**Additional Information:**

Please access these Web sites via the online version of this summary at http://dx.doi.org/10.1371/journal.pmed.1000354.

- This study is further discussed in a *PLoS Medicine* Perspective by Harvey Marcovitch
- The International Committee of Medical Journal Editors provides information about the publication of medical research, including conflicts of interest
- The World Association of Medical Editors also provides information on conflicts of interest in medical journals
- Information about impact factors is provided by Thomson Reuters, a provider of intelligent information for businesses and professionals; Thomson Reuters also runs Web of Science

## Introduction

Many medical journals require that authors and peer reviewers declare whether they have any conflicts of interest. Such knowledge can be important for readers when assessing the paper and for editors when assessing the peer review comments.

Editors can also have conflicts of interest, and the International Committee of Medical Journal Editors states that: “Editors who make final decisions about manuscripts must have no personal, professional, or financial involvement in any of the issues they might judge” [1]. Furthermore, editors are advised to “publish regular disclosure statements about potential conflicts of interest related to the commitments of journal staff” [1].

Journals may have other conflicts of interest than those of their editors, and the most important of these is likely related to the publication of industry-supported clinical trials. It is important for the industry to publish reports of large trials in prestigious journals, as such reports are essential for clinical decision making and for the sales of drugs and devices [2],[3]. However, journals not only stand to gain financially through the sales of reprints, but also publication of such trials may increase their impact factors, as a large number of reprints distributed to key clinicians by drug companies will likely increase citation rates. For example, *The New England Journal of Medicine* (*NEJM*) sold about one million reprints to Merck of its paper on the VIGOR trial of rofecoxib [4].

One survey found that the policies on conflicts of interest for individual editors vary between journals [5], but we have not been able to identify any empirical studies on conflicts of interest at medical journals.

We investigated the influence of industry-supported randomised trials on impact factors for major general medical journals and describe the relative income from the sales of advertisements, reprints, and industry-supported supplements.

## Methods

The impact factor for a given year is calculated as the number of citations in that year to papers published in the two previous years, divided by the number of citable papers published in the two previous years [6]. We focused on two time periods, citations in 1998 for randomised clinical trials (RCTs) published in 1996–1997 and citations in 2007 for RCTs published in 2005–2006, and retrieved citation data using Web of Science on the ISI Web of Knowledge [7].

On the basis of a pilot (see Text S1) that included journals categorised as “Medicine, General & Internal” in Journal Citation Reports on the ISI Web of Knowledge [8] with an impact factor of five or higher in 2007, which identified ten journals, we decided to include six major general medical journals: *Annals of Internal Medicine* (*Annals*), *Archives of Internal Medicine* (*Archives*), *BMJ*, *JAMA*, The *Lancet* (*Lancet*), and *NEJM*. Trials published in these journals in the two time periods were identified using PubMed's limits function for journal name, publication date, and the publication type Randomized Controlled Trial. Papers that were not full reports of trials (e.g., letters, commentaries, and editorials) were excluded. We extracted information on journal name, title, publication year, and type of support into a standardised data sheet. Data extraction and retrieval of citation data were done independently by two authors (AL, MB), and discrepancies were resolved by discussion.

### Type of Support

We categorised the support as industry support, mixed support, nonindustry support, or no statement about support. We defined industry support as any financial support, whether direct or indirect (e.g., grants, industry-employed authors, assistance with data analysis or writing of the manuscript, or provision of study medication or devices by a company that produces drugs or medical devices). We did not regard a study as industry-supported if the only interaction with industry was author conflicts of interest (e.g., honorariums, consultancies, and membership of advisory boards). We defined nonindustry support as any other type of financial support. Mixed support was any support provided by both industry and nonindustry sources, and no statement about support if nothing was stated in the paper.

### Citations of Individual Trials and of All Papers

We identified the number of citations for each identified RCT using the function “refine by publication year” in Web of Science. This was done in July and August 2009, blinded to the study support of the individual trials. We also identified the total number of citations (the numerator of the impact factor) using the function “create citation report” in Web of Science.

In our pilot study we compared the number of citations using Web of Science with the numbers used for the “official” impact factor calculation published in Journal Citation Reports and we discovered that the numbers of citations in Web of Science were lower (see Text S1). Correspondence with the publisher, Thomson Reuters, revealed that the citations from Web of Science and Journal Citation Reports are not similar, as they are not based on the same data. For example, studies that are referenced incorrectly are only included in Journal Citation Reports. But determinants leading to a citation not being identified in Web of Science can be assumed to be random and unrelated to study type and support. As our aim was to look at the relative contribution of industry-supported trials to the impact factor, we proceeded with our planned analyses, calculating an approximate impact factor.

### Denominator of Impact Factor

We identified the number of citable papers (denominator of impact factor) published in 1996–1997 and 2005–2006 for each journal using the most recent Journal Citation Report with available data.

### Financial Income and Reprint Sales

In November 2009, we contacted the Editor-in-Chief of each of the six journals by e-mail and requested data on income from sales of advertisements, reprints, and industry-supported supplements (if any), as percentage of total income for the journal, and the total number of reprints sold, in both cases in 2005 and 2006.

*BMJ* and *Lancet* provided the data, but the editors of Archives, *JAMA*, and *NEJM* did not provide the data, as it was their policy not to disclose financial information. *Annals* forwarded our request to the publisher who declined for similar reasons.

For these four American journals, we therefore needed to use proxy data. We obtained the publicly available tax information stated in the Internal Revenue Service Form 990 (tax form required for nonprofit organizations) for 2005 and 2006 for the journal owners: American College of Physicians (ACP) for *Annals*, American Medical Association (AMA) for *Archives* and *JAMA*, and Massachusetts Medical Society (MMS) for *NEJM*. These data report on the total income from all types of publishing by the societies, and as all societies publish more than one journal, we could not obtain data for individual journals. ACP publishes three other journals in the ACP series and books; AMA publishes eight other journals in the Archives series, an additional journal, and Web-based material; and MMS publishes various article summaries in their Journal Watch series. We contacted the journal owners for confirmation of our calculations for the relative income from industry sources based on tax information. ACP confirmed our calculations, but AMA and MMS did not reply, despite numerous e-mails.

### Statistical Analyses

For each journal, we compared the distribution of support of trials published in the two time periods with the Mann-Whitney U-test (two-sided). In an a priori stated sensitivity analysis, we recategorised trials with no statement about support as nonindustry supported.

On the basis of our own citation data derived from the individual trials, we compared the number of citations of trials with industry support with those with mixed support and those with nonindustry support using the Jonckheere-Terpstra test for trend (two-sided). As the category “not stated” is a mix of the three other categories we did not include this in our test. To test the robustness of our findings, we did various a priori stated sensitivity analyses (e.g., change in criteria used for industry support) (see Text S1).

We calculated what an approximate impact factor would have been, for each of the six journals for each time period, if no trials with industry support had been published; this calculation was done by excluding trials with industry support from the numerator and the denominator. We did the same calculations using a broader category of industry-supported trials that also included those with mixed support. We estimated the percentage reduction in impact factor that resulted from exclusion of these trials.

We had intended to study the association between mean number of citations to trials and percent income from reprints, but this was not possible as only the two European journals provided the data we requested.

## Results

We identified 1,429 papers indexed as Randomized Controlled Trials in PubMed (see Text S1) and excluded 61 letters, three editorials, five commentaries, and seven papers that were e-published ahead of print, which yielded a total sample size of 1,353 included RCTs (651 from 1996–1997 and 702 from 2005–2006).

For *Annals* and *Lancet*, the number of trials decreased over time, whereas it increased for the other journals (see Table 1). The total number of citable papers decreased for all journals, except *Archives*. Hence, there was an increase in the proportion of trials out of all citable papers for all journals over time, with *BMJ* and *JAMA* having around a 3-fold increase and *NEJM* having the highest proportion of trials in both periods.

**Table 1:** Description of support of randomised controlled trials published in major general medical journals.

Support	*Annals*		*Archives*		*BMJ*		*JAMA*		*Lancet*		*NEJM*	
	1996–1997	2005–2006	1996–1997	2005–2006	1996–1997	2005–2006	1996–1997	2005–2006	1996–1997	2005–2006	1996–1997	2005–2006
*n* trials (%)	71 (16)	58 (17)	67 (13)	80 (13)	91 (6)	116 (16)	76 (7)	113 (19)	186 (12)	129 (20)	160 (20)	206 (34)
Total *n* citable papers	458	339	519	593	1624	745	1120	590	1515	661	785	611
**Support of trials**												
Industry support (%)	19 (27)	11 (19)	22 (33)	12 (15)	12 (13)	8 (7)	23 (30)	29 (26)	47 (25)	28 (22)	51 (32)	66 (32)
Mixed support (%)	27 (38)	20 (34)	14 (21)	23 (29)	19 (21)	23 (20)	21 (28)	33 (29)	46 (25)	46 (36)	54 (34)	95 (46)
Nonindustry support (%)	19 (27)	27 (47)	24 (36)	37 (46)	52 (57)	82 (71)	26 (34)	50 (44)	61 (33)	55 (43)	42 (26)	41 (20)
Not stated (%)	6 (8)	0 (0)	7 (10)	8 (10)	8 (9)	3 (3)	6 (8)	1 (1)	32 (17)	0 (0)	13 (8)	4 (2)
Change in support (*p*-value)*	—	0.047	—	0.041	—	0.101	—	0.255	—	0.251	—	0.498

*Comparison of number of trials with industry, mixed, and nonindustry support in 1996–1997 versus 2005–2006 using Mann-Whitney U-test (two-sided).

### Type of Support

The type of support varied markedly across journals (see Table 1). In 2005–2006, *NEJM* had the highest proportion of trials with industry support (32%) and *BMJ* the lowest (7%). The proportion of trials that were industry-supported declined from 1996 to 2005 for all journals except *NEJM*, where it was constant. The decline was statistically significant for *Annals* and *Archives*; for *BMJ*, the proportion declined by nearly half (from 13% to 7%), but this was not statistically significant.

### Citations of Individual Trials

For trials published in 1996–1997, there was a significant relation between the number of citations and the degree of industry support (three categories on a ranking scale) for *Lancet* (*p* = 0.003) and *NEJM* (*p* = 0.003), whereas for trials published in 2005–2006, the relation was statistically significant for all journals (see Table 2). Industry-supported trials published in *Annals*, *Archives*, and *Lancet* in 2005–2006 were cited more than twice as often as nonindustry trials, and one and a half times more in *BMJ*, *JAMA*, and *NEJM*.

**Table 2:** Citations for randomised trials published in major general medical journals and change in impact factors when industry-supported trials are excluded.

Citation and Impact Factor	*Annals*		*Archives*		*BMJ*		*JAMA*		*Lancet*		*NEJM*	
	1996–1997	2005–2006	1996–1997	2005–2006	1996–1997	2005–2006	1996–1997	2005–2006	1996–1997	2005–2006	1996–1997	2005–2006
**Mean ** ***n*** ** citations** a												
Industry support	13.3	27.7	6.5	14.4	7.6	9.9	21.3	35.7	30.4	53.5	58.1	82.2
Mixed support	18.7	17.1	9.4	11.8	7.3	11.3	20.1	31.6	31.5	31.3	46.9	66.7
Nonindustry support	9.3	12.0	5.5	6.5	7.3	5.7	13.8	21.1	14.0	25.7	34.5	47.3
Not stated	10.8	—	10.9	8.3	8.0	5.0	5.7	32.0	10.0	—	33.4	30.3
Difference in citations (*p*-value)^b^	0.186	≪0.001	0.237	≪0.001	0.949	0.033	0.115	0.011	0.003	0.016	0.003	≪0.001
**Change in impact factor**												
Without trials with industry support (%)	−1	0	−1	−2	0	0	−3	−3	−5	−4	−7	−7
Without trials with industry and mixed support (%)	−6	−4	−3	−4	−1	−1	−5	−5	−11	−6	−13	−15

a For each journal citations are reported for their impact factor year (i.e., citations in 1998 to trials published in 1996–1997 and in 2007 to trials published in 2005–2006).

b Difference in citations depending on type of support using Jonckheere-Terpstra test for trend (two-sided, support not stated excluded from the analysis).

Our a priori defined sensitivity analyses found minor discrepancies, but overall the results were robust (see Text S1).

### Approximate Impact Factor

The approximate impact factor we calculated decreased for all journals when industry-supported trials were excluded from the calculation, and the decrease was larger when trials with mixed support were also excluded (see Table 2). The decrease was highest for *NEJM*, followed by *Lancet*, whereas the impact factor of *BMJ* was barely affected. The decrease in approximate impact factor varied minimally between the two time periods for all journals, except for *Lancet*, where the decrease was 11% in 1996–1997 and 6% in 2005–2006 when trials with industry and mixed support were excluded.

### Financial Income and Reprint Sales

In 2005–2006, 16% of the income for *BMJ* was from display advertisements, 3% from reprints, and 0% from supplements, and 967,930 reprints were sold (see Table 3). For *Lancet*, the percentages were 1% from display advertisements, 41% from reprints, and 0% from supplements, and 11,514,137 reprints were sold. For ACP, the Internal Revenue Service data did not specify the income, for AMA 53% of the income was from advertisements and 12% from reprints (no data on supplements), and for MMS 23% of the income was from advertisements, whereas there were no data on reprints and supplements.

**Table 3:** Relative income of journals and medical societies from sales of advertisements, reprints, and supplements and number of reprints sold in 2005–2006.

Income and *n* Reprints Sold	*BMJ*	*Lancet*	*ACP* (*Annals*)	*AMA* (*Archives* and *JAMA*)	MMS (*NEJM*)
Advertising (%)	16	1	Not stated	53	23
Reprints (%)	3	41	Not stated	12	Not stated
Supplements (%)	0	0	Not stated	Not stated	Not stated
*n* reprints sold	967,930	11,514,137	Not stated	Not stated	Not stated

## Discussion

We found that the proportion of industry-supported trials varied widely across journals but changed very little for each journal within the studied time period. Industry-supported trials boosted the approximate impact factor we calculated for all six journals—the most for *NEJM* and the least for *BMJ*. Only the two European journals disclosed their main sources of income, and the income from selling reprints was hugely different, as it comprised 41% of the total income for *Lancet* and only 3% for *BMJ*.

We believe this is the first study that investigates potential conflicts of interest at medical journals in relation to citations and financial income from publication of industry-supported trials. Our data collection was systematic and thorough, but there are also limitations. First, we selected major general medical journals that publish many trials, and our findings may therefore not be generalisable to other journals. Second, our assumption that trials with no statement of support, or with nonindustry support, were not industry supported may have led to an underestimation, as undeclared industry involvement is common, e.g., in relation to ghost authorship by medical writers' agencies [9]. However, the proportion of trials with no statement of support was very small in the second period. Third, owing to the nature of the Web of Science database, our identified citations were not the same as those used when calculating journal impact factors in the Journal Citation Reports. But as our aim was to study the relative influence of industry-supported trials on the impact factor, this discrepancy is likely not so important, as errors in citing studies would be expected to be unrelated to the type of support they received.

A problem related to the impact factor is that so-called noncitable papers such as editorials, news pieces, and letters to the editor contribute to the numerator, but not to the denominator [10],[11]. Because of this serious deficiency in the calculation, we believe we have underestimated considerably the true influence of industry-supported trials on the approximate impact factor. For example, if we only include citations to citable papers (i.e., original research and reviews) in an analysis of trials with industry and mixed support, we find that the 2007 impact factor of *NEJM* decreases by 24% instead of 15%.

There are several reasons why industry-supported trials are generally more cited than other trials and other types of research. They are often large and the most used interventions are drugs, which are known to increase citations [12],[13]. One explanation might be that industry-supported trials are of higher quality than nonindustry supported trials, though there is little evidence to support this [14]. Interestingly, industry trials more often have positive results than nonindustry trials [14], the conclusions in negative trials are often presented in such a way that they appear to be more positive than they actually are [15], and positive industry trials are more cited than negative ones [12],[13]. Furthermore, sponsoring companies may employ various strategies to increase the awareness of their studies, including ghost authored reviews that cite them [16]–[19], purchase and dissemination of reprints [20], and creation of media attention [12],[21],[22]. Such strategies are likely to be predominantly used for trials favourable to the sponsors' products, and this may put editors under pressure, as they know which papers are especially attractive for the companies [3].

Editors have an interest in increasing the impact factor for their journal [23], whereas journal finances are generally regarded to be in the hands of the publisher. However, editors also have an interest in this, as they might be forced to fire staff if the journal does not remain profitable, or at least viable. The former editor of the *BMJ*, Richard Smith, reported that a single trial may lead to an income of US$1 million for a journal from reprint sales [21], and with a large profit margin of around 70% [3]. Journal publishers therefore have an incentive to advertise the benefits of reprints. As examples, the BMJ Group states that “Medical specialists determine the success of your product or service. They influence teaching, practice and purchase decisions within their workplace and the whole specialty. Reach them through the BMJ Group reprints service” [24], and *NEJM* states that “Article reprints from the New England influence treatment decisions” [25]. The editor of The *Lancet*, Richard Horton, has described how companies sometimes offer journals to purchase a large number of reprints and may threaten to pull a paper if the peer review is too critical [26]. For *Lancet*, a little less than half of the journal income came from reprints, and though this applied to only 12% of *AMA*'s income, it could be more for its most prestigious journal, *JAMA*. Large incomes from reprint sales would also be expected for *NEJM*, as it publishes more industry-supported trials than the other journals.

This area warrants further research. Speciality journals could also be investigated, particularly as conflicts of interest there could be more pronounced as editors are often investigators themselves and the degree of industry support may vary across specialities. Influence from other types of industry-sponsored papers could also be investigated. It would also be interesting to examine whether income from advertisements can affect editorial decisions; for example, one could compare the number of advertisements from specific companies with the number of publications from the same companies in a sample of journals. Such income can be substantial for some journals [27]. When *Annals* published a study that was critical of industry advertisements [28], it resulted in the loss of an estimated US$1–1.5 million in advertising revenue [29]. Another source of income that should be investigated is sponsored subscriptions whereby companies pay for subscriptions for clinicians, sometimes with a cover wrap displaying a company product [30].

Although publication of industry-supported trials is favourable for medical journals we cannot tell from our results whether industry-supported trials have affected editorial decisions. The high number of industry-supported trials in *NEJM* could merely result from the fact that it has the highest impact factor and therefore also receives more trial reports than other journals. Nevertheless, disclosure of conflicts of interest is not about whether relationships have actually influenced decisions, but whether they potentially could have [1]. The International Committee of Medical Journal Editors requires authors to “disclose interactions with ANY entity that could be considered broadly relevant to the work” [31]. We suggest that journals abide by the same standards related to conflicts of interest, which they rightly require from their authors, and that the sources and the amount of income are disclosed to improve transparency.

## Supporting Information

Text S1 Appendix. Additional information on the methods used and results of study inclusion and sensitivity analyses.
(0.06 MB DOC)

## Abbreviations

ACP: American College of Physicians
AMA: American Medical Association
MMS: Massachusetts Medical Society
RCT: randomised controlled trial
