# Correction: Conflicts of Interest at Medical Journals: The Influence of Industry-Supported Randomised Trials on Journal Impact Factors and Revenue – Cohort Study

**DOI:** 10.1371/annotation/7e5c299c-2db7-4ddf-8eff-ab793511eccd

**Published:** 2011-02-25

**Authors:** Andreas Lundh, Marija Barbateskovic, Asbjørn Hróbjartsson, Peter C. Gøtzsche

The Academic Editor's name and institution were not included on the PDF or HTML versions of this article in error.

The Academic Editor providing expert input on this paper was Harvey Marcovitch, ex-Chairman of the Committee on Publications Ethics (http://publicationethics.org/) and currently Editor in Chief of Clinical Risk, RSM Press Ltd., London, United Kingdom. For more information about the role of the Academic Editor in PLoS Medicine's editorial process, see our Author Guidelines: http://www.plosmedicine.org/static/guidelines.action#overview

Harvey Marcovitch is also the author of a related Perspective article discussing this research. 

