# Supplementary material for: Conflicts of Interest at Medical Journals: The Influence of Industry-Supported Randomised Trials on Journal Impact Factors and Revenue – Cohort Study
Source: PLoS Med. 2010 Oct 26;7(10):e1000354. doi: 10.1371/journal.pmed.1000354 (PMC2964336; doi:10.1371/journal.pmed.1000354)
Supplement: Text S1 — Appendix. Additional information on the methods used and results of study inclusion and sensitivity analyses. (0.06 MB DOC) [file pmed.1000354.s001.doc]

**Additional Information**

**Methods**

Journal Selection

In order to obtain a sample of major general medical journals publishing randomised clinical trials (RCTs), we chose journals categorised as “Medicine, General & Internal” in Journal Citation Reports on the ISI Web of Knowledge (1) with an impact factor of five or higher in 2007 and identified 10 journals. To ensure similarities for comparison across journals, we excluded Annual Review of Medicine, as it is only published once a year and does not publish RCTs. PLoS Medicine, Canadian Medical Association Journal and Annals of Medicine were also excluded, as only 4/166, 5/94 and 0/54, respectively, of their papers in 2007 were indexed in PubMed as the publication type Randomized Controlled Trial, which we regarded as too low a proportion of RCTs for our study. Our final inclusion consisted of six journals: Annals of Internal Medicine (Annals), Archives of Internal Medicine (Archives), BMJ, JAMA, The Lancet (Lancet) and The New England Journal of Medicine (NEJM).

Pilot study

In a pilot study done in December 2008, one author (AL) extracted data on support for all randomised trials published in NEJM in 2000 and 2001. Using Web of Science on the ISI Web of Knowledge (2) (see method below) the mean number of citations in 2002 was 82.1 for RCTs with industry support, 50.9 for RCTs with mixed support, 36.3 for RCTs with non-industry support and 26.9 for RCTs with no statement about support (p < 0.0001 for difference; Jonckheere-Terpstra test for trend). The impact factor for 2002 was lowered by 11% when industry-supported RCTs were excluded from the calculation, and by 17% if RCTs with mixed support were also excluded (see method below).

Citation data

Based on the citation data from Web of Science we tried to recalculate the impact factor for 2002 for the New England Journal of Medicine in. This led to an impact factor that was 9% lower than the ‘official’ impact factor reported in the Journal Citation Report for 2002.

Further exploration and correspondence with Thomson Reuters (the publisher of Web of Science) led us to the conclusion that it was impossible to recalculate the exact impact factor for a given journal using data available in Web of Science. First, the data in the database are not static, but new data are added and old data are corrected. Therefore, the data used to calculate the impact factor vary over time. Second, some citations are erroneous (e.g. wrong page number or issue) and are therefore not linked to the correct study. These citations will be missed when searching Web of Science, as it only identifies the properly linked studies, but they are used for calculating the impact factor in the Journal Citation Reports. Third, the only option available for getting data for total citations for a given journal in a given year (numerator of impact factor) is the “create citation report” function in Web of Science. Unfortunately, this method uses the ‘date-of-entry’ of the citation into the database and not the ‘date of publication’. We requested data using ‘date of publication’ from Thomson Reuters, but were told that it would cost us 4,500 US $ to obtain the data. Due to these costs and because editors have complained about the quality of these data (3,4) we did not pursue this matter further.

Due to the discrepancies between the data in Journal Citation Reports and Web of Science encountered in our pilot, we also extracted the total number of citations for the six different journals for each year from 1998 to 2007. Data from Web of Science gave between 80% and 97% of the citations in Journal Citation Reports depending on the specific journal and year.

Sensitivity analysis

To test the robustness of our support data we did an a priori stated sensitivity analysis where we re-categorised RCTs with no statement about support as non-industry supported.

To test the robustness of our citation data, we did three a priori stated sensitivity analyses. First, we re-categorised RCTs with no stated support as non-industry support. Second, we re-categorised RCTs where the only support was free study drugs or devices as mixed instead of industry support. Third, we calculated the mean number of citations based on the function “create citation report” in Web of Science. This strategy uses the ‘date-of-entry’ of the citation rather than the ‘date of publication’.

**References**

1) ISI Web of Knowledge. Journal Citation Reports. Available: [http://www.isiknowledge.com](http://www.isiknowledge.com/) Accessed 11 March 2010.

2) ISI Web of Knowledge. Web of Science. Available: [http://www.isiknowledge.com](http://www.isiknowledge.com/) Accessed 11 March 2010.

3) Rossner M, Van Epps H, Hill E (2007) Show me the data. J Cell Biol 179:1091-2.

4) [no authors] (2006) The impact factor game. It is time to find a better way to assess the scientific literature. PLoS Med 3:e291.

Description of included randomised trials.

| **Journal name** | **Year**  **published** | **Identified**  **papers** | **Excluded**  **papers** | **Included**  **trials** |
| --- | --- | --- | --- | --- |
| Annals | 1996-7 | 73 | 2 letters | 71 |
|  | 2005-6 | 59 | 1 paper* | 58 |
|  |  |  |  |  |
| Archives | 1996-7 | 68 | 1 letter | 67 |
|  | 2005-6 | 81 | 1 editorial | 80 |
|  |  |  |  |  |
| BMJ | 1996-7 | 103 | 9 letters  2 editorials  1 commetary | 91 |
|  | 2005-6 | 122 | 6 papers* | 116 |
|  |  |  |  |  |
| JAMA | 1996-7 | 76 |  | 76 |
|  | 2005-6 | 113 |  | 113 |
|  |  |  |  |  |
| Lancet | 1996-7 | 234 | 44 letters  4 commentaries | 186 |
|  | 2005-6 | 129 |  | 129 |
|  |  |  |  |  |
| NEJM | 1996-7 | 163 | 3 letters | 160 |
|  | 2005-6 | 208 | 2 letters | 206 |

* Papers were excluded because they were only e-published in 2006, but published in the journal in 2007 and indexed in Web of science as a 2007 publication.

Sensitivity analysis for type of support of randomised controlled trials published in major general medical journals when no statement about support was categorised as non-industry.

|  | Annals | | Archives | | BMJ | | JAMA | | Lancet | | NEJM | |
| --- | --- | --- | --- | --- | --- | --- | --- | --- | --- | --- | --- | --- |
|  | 1996-7 | 2005-6 | 1996-7 | 2005-6 | 1996-7 | 2005-6 | 1996-7 | 2005-6 | 1996-7 | 2005-6 | 1996-7 | 2005-6 |
| Change in support (p value)* | - | 0.164* | - | 0.063* | - | 0.191* | - | 0.551* | - | 0.544* | - | 0.130* |

* Comparison of number of RCTs with industry, mixed and non-industry support in 1996-7 vs. 2005-6 using Mann-Whitney U test (two sided).

Sensitivity analysis for citations for randomised controlled trials published in major general medical journals when no statement about support was categorised as non-industry supported, when RCTs with free drug provision as only type of support were categorised as mixed and when citation data was based on “create citation report”.

|  | Annals | | Archives | | BMJ | | JAMA | | Lancet | | NEJM | |
| --- | --- | --- | --- | --- | --- | --- | --- | --- | --- | --- | --- | --- |
|  | 1996-7 | 2005-6 | 1996-7 | 2005-6 | 1996-7 | 2005-6 | 1996-7 | 2005-6 | 1996-7 | 2005-6 | 1996-7 | 2005-6 |
| Difference in citations - “not stated” (p-value)# | 0.096 | << 0.001 | 0.316 | < 0.001 | 0.892 | 0.030 | 0.023 | 0.013 | <<0.001 | 0.016 | 0.001 | <0.001 |
| Difference in citations - “free drug” (p-value)* | 0.115 | << 0.001 | 0.205 | < 0.001 | 0.818 | 0.030 | 0.070 | 0.011 | 0.003 | 0.016 | 0.002 | <0.001 |
| Difference in citations - “create citation report” (p-value)$ | 0.212 | << 0.001 | 0.232 | < 0.001 | 0.912 | 0.082 | 0.118 | 0.002 | 0.004 | 0.007 | 0.003 | <0.001 |

# Difference in citations depending on type of support using Jonckheere-Terpstra test for trend (two sided).

* Difference in citations depending on type of support using Jonckheere-Terpstra test for trend (two sided).

$ Difference in citations depending on type of support using Jonckheere-Terpstra test for trend (two sided).
